# Commentary: Blood metabolites as mediators in erectile dysfunction: insights from a multi-center proteomics and genetic study

**DOI:** 10.3389/fphar.2025.1650398

**Published:** 2025-09-04

**Authors:** Zhihao Huang, Hongxing Gu

**Affiliations:** Department of Urology, Zhangjiagang Hospital Affiliated to Soochow University, Suzhou, Jiangsu, China

**Keywords:** erectile dysfunction, blood metabolites, multi-center proteomics, genetic study, commentary

With great interest, I read the article by Chen et al. (2025a), recently published in Frontiers in Pharmacology. This study employs Mendelian randomization (MR) analysis, combined with a multicenter proteomics database and the Finnish FinnGen database, to identify circulating proteins that have a causal association with erectile dysfunction (ED) has successfully identified five plasma proteins (AMN, ESM1, KIR2DL2, PIGR, TNFRSF6B) demonstrating causal associations with ED. This study significantly advances ED biomarker research. Here, we propose methodological refinements to strengthen the clinical implications of these findings.

First, I noticed that the identified biomarkers may potentially exhibit collinearity due to their involvement in shared inflammatory or metabolic pathways. ESM1, a known regulator of angiogenesis and vascular permeability, has been shown to be overexpressed in diabetic ED patients, correlating strongly with endothelial dysfunction (Balta and Mikhailidis, 2019). For instance, ESM1 and TNFRSF6B both modulate endothelial function——a key pathway in ED pathogenesis (Xu et al., 2021). To evaluate independence of effects, we suggest that performing multivariable MR analysis while adjusting for potential confounders such as diabetes and obesity status could help determine whether these proteins independently contribute to ED risk. Recent methodological advances highlight the necessity to address sample overlap and heterogeneity when integrating multiple GWAS sources. As demonstrated in the critical appraisal by Chen et al. (2025b) for myocardial infarction protein targets, unbalanced meta-analysis of genetically correlated traits may inflate errors. We recommend applying genomic relationship matrix based approaches to correct for sample overlap between FinnGen and UK Biobank cohorts.

Additionally, the FinnGen database (Release 10, comprising 391,037 controls) provides substantial statistical power, its exclusive focus on Finnish ancestry may introduce population-specific biases. To enhance the generalizability of these findings, we recommend conducting a comprehensive meta-analysis incorporating: The UK Biobank dataset (with ED phenotypes defined by ICD-10 codes F52 and N48.4) (Nagar et al., 2023), Large-scale genetic consortia such as the Psychiatric Genomics Consortium (PGC) and Social Science Genetic Association Consortium (SSGAC), which include more diverse ancestral populations.

Finally, we believe that the study would benefit from experimental validation of the identified biomarkers in ED pathophysiology. Our literature review reveals several important considerations: The Mendelian randomization analysis revealed a positive association between AMN and ED risk, which presents a potential conflict with established renal physiology (Kozyraki et al., 2022). In the renal system, AMN deficiency causes Imerslund-Gräsbeck syndrome, while its overexpression in circulation lacks clear pathological characterization (Salvio et al., 2021). Animal models demonstrate that AMN knockout reduces cubilin-mediated protein reabsorption while increasing circulating homocysteine levels, the latter being a known ED risk factor (Pannérec et al., 2018). Its circulatory role in ED may involve homocysteine elevation. This duality warrants further study. The study’s findings of ESM1 upregulation and TNFRSF6B downregulation suggest novel mechanisms in vascular homeostasis. Notably, its hypoxia-induced downregulation in tissues (Zheng et al., 2022) contrasts with the MR-predicted upregulation in ED plasma, suggesting a compensatory circulating response. Similarly, TNFRSF6B—a decoy receptor for Fas ligand—modulates endothelial apoptosis. Its downregulation in ED may reflect impaired vascular repair, though tissue-specific studies are needed to confirm this mechanism. Although KIR2DL2 is primarily involved in NK-cell activity modulation, and inflammation is recognized as a contributor to ED, its association with ED could reflect inflammation, but penile tissue studies are lacking (Moradi et al., 2021). Normally, decreased KIR family protein expression releases NK cell inhibition, promoting inflammation (Zuo et al., 2022). For PIGR, while typically downregulated during systemic inflammation as a mucosal immunity protein, it shows paradoxical upregulation in diabetic endothelium (Wei and Wang, 2021). Its upregulation in ED might indicate mucosal barrier disruption in diabetic endothelium. This context-dependent expression necessitates refined subgroup analyses. For human validation, we will analyze: Serum samples from 50 ED patients (25 diabetic/25 non-diabetic) For mechanistic studies, we will employ: High-fat diet-induced ED mouse models (n = 40).

We commend the authors for their important contribution to the field of sexual medicine, the identified biomarkers show diagnostic promise, we believe these additional investigations would further solidify the foundation for precision medicine approaches to ED treatment.

## References

[B1] Balta S. Mikhailidis D. P. (2019). Endocan and erectile dysfunction. Am. J. men's health 13 (6), 1557988319893889. 10.1177/1557988319893889 31829075 PMC6909267

[B2] Chen J. Zhao J. Zhang Z. Zhu X. Zuo J. Nie Z. (2025a). Blood metabolites as mediators in erectile dysfunction: insights from a multi-center proteomics and genetic study. Front. Pharmacol. 16, 1568780. 10.3389/fphar.2025.1568780 40529500 PMC12171135

[B3] Chen J. Zhao J. Fu S. (2025b). Addressing sample overlap and meta-analysis imbalances in Mendelian randomization: a critical appraisal of protein targets for myocardial infarction. Eur. heart J. 46. 2928–2929. 10.1093/eurheartj/ehaf186 40249360

[B4] Kozyraki R. Verroust P. Cases O. (2022). Cubilin, the intrinsic factor-vitamin B12 receptor. Vitamins hormones 119, 65–119. 10.1016/bs.vh.2022.01.005 35337634

[B5] Moradi S. Stankovic S. O'Connor G. M. Pymm P. MacLachlan B. J. Faoro C. (2021). Structural plasticity of KIR2DL2 and KIR2DL3 enables altered docking geometries atop HLA-C. Nat. Commun. 12 (1), 2173. 10.1038/s41467-021-22359-x 33846289 PMC8041999

[B6] Nagar S. D. Jordan I. K. Mariño-Ramírez L. (2023). The landscape of health disparities in the UK Biobank. Database J. Biol. databases curation 2023, baad026. 10.1093/database/baad026 37114803 PMC10132819

[B7] Pannérec A. Migliavacca E. De Castro A. Michaud J. Karaz S. Goulet L. (2018). Vitamin B12 deficiency and impaired expression of amnionless during aging. J. cachexia, sarcopenia muscle 9 (1), 41–52. 10.1002/jcsm.12260 29159972 PMC5803611

[B8] Salvio G. Ciarloni A. Cutini M. Balercia G. (2021). Hyperhomocysteinemia: focus on endothelial damage as a cause of erectile dysfunction. Int. J. Mol. Sci. 22 (1), 418. 10.3390/ijms22010418 33401548 PMC7795368

[B9] Wei H. Wang J. Y. (2021). Role of polymeric immunoglobulin receptor in IgA and IgM transcytosis. Int. J. Mol. Sci. 22 (5), 2284. 10.3390/ijms22052284 33668983 PMC7956327

[B10] Xu S. Ilyas I. Little P. J. Li H. Kamato D. Zheng X. (2021). Endothelial dysfunction in atherosclerotic cardiovascular diseases and beyond: from mechanism to pharmacotherapies. Pharmacol. Rev. 73 (3), 924–967. 10.1124/pharmrev.120.000096 34088867

[B11] Zheng X. Higdon L. Gaudet A. Shah M. Balistieri A. Li C. (2022). Endothelial cell-specific Molecule-1 inhibits albuminuria in diabetic mice. Kidney360 3 (12), 2059–2076. 10.34067/KID.0001712022 36591362 PMC9802554

[B12] Zuo W. Yu X. X. Liu X. F. Chang Y. J. Wang Y. Zhang X. H. (2022). The interaction of HLA-C1/KIR2DL2/L3 promoted KIR2DL2/L3 Single-Positive/NKG2C-Positive natural killer cell reconstitution, raising the incidence of aGVHD after hematopoietic stem cell transplantation. Front. Immunol. 13, 814334. 10.3389/fimmu.2022.814334 35572602 PMC9101514

